# C-753-08. Utilizing AI Decision Support Tools in Burn Resuscitation: Comparing Large Language Models to Burn Navigator

**DOI:** 10.1093/jbcr/irag033.028

**Published:** 2026-04-06

**Authors:** Shawn Tejiram, Tamer R Hage, Alina A Watson, Atharva Bendre, Tuan D Le, Bonnie C Carney, Lauren T Moffatt, Jeffrey W Shupp

**Affiliations:** MedStar Washington Hospital Center, Washington, DC; Virginia Commonwealth University, Arlington, VA; Georgetown University, Washington, DC; University of Virginia, Charlottesville, VA; The Burn Center at MedStar Washington Hospital Center, Washington, DC; The Burn Center at MedStar Washington Hospital Center, Washington, DC; The Burn Center at MedStar Washington Hospital Center, Washington, DC; MedStar Washington Hospital Center, Washington, DC

## Abstract

**Introduction:**

Crystalloid is the mainstay for treatment following acute severe burn injury. Traditional approaches for fluid resuscitation rely on calculated formulae, close monitoring, and titration based on end points of resuscitation to avoid complications. In recent years, computerized Clinical Decision Support (CDS) systems, such as the Burn Navigator (BN) platform, have automated this process and improved goal-directed outcomes. However, widespread use remains limited and may in part be due to cost and accessibility. Emerging artificial intelligence (AI) tools such as Large Language Models (LLMs) offer cost-effective data-driven alternatives. The aim of this work was to explore the use of an LLM to guide burn resuscitation recommendations. This study sought to compare fluid resuscitation estimates between a commonly used LLM and BN.

**Methods:**

A retrospective review over one year was conducted on adult patients with total body surface area (TBSA) burn size of 20% or greater at an ABA verified burn center who received individualized fluid resuscitation guided by BN during the first 24 hours after injury. An LLM (Chat GPT 5, accessed through the OpenAI Plus subscription plan) was given a series of standardized prompts to query for recommended hourly crystalloid infusion rates based on identical clinical inputs used by BN, including age, TBSA, weight, preferred resuscitation formulae, time of injury, urine output, and relevant confounders (e.g., inhalation or electrical injury). Bland–Altman analysis was used to compare fluid volume predictions between the LLM and BN using BN-generated hourly fluid rate recommendations as the reference standard. Differences between methods were plotted against their mean values to assess bias and limits of agreement.

**Results:**

Eighteen patients [mean (SD) TBSA of 41% (23.2%)] contributed a total of 387 fluid rate recommendations. Mean fluid rates estimated by the LLM and BN showed no statistically significant difference [462 (430-494) mL/hr vs 450 (421-479) mL/hr, *p*=.57]. Pearson correlation analysis demonstrated a strong linear relationship between the two methods (r = 0.93; 95% CI, 0.91-0.94, *p*<.0001). Bland–Altman analysis revealed strong agreement, with a mean difference [12.3 mL/hr (95% CI, -222 to 246 mL/hr)] and limits of agreement ranging from -400 to 600 mL/hr. Approximately 95% of data points fell within 200 mL/hr of the mean difference. Larger discrepancies were observed most at fluid rates of 500 mL/hr to 1000 mL/hr.

**Conclusions:**

LLMs demonstrate potential as a scalable CDS tool in burn resuscitation and demonstrated overall agreement with BN. Some variability in moderate fluid rates highlight the need for further refinement and clinical validation. LLMs may be considered complementary to established platforms.

**Applicability of Research to Practice:**

This comparison of LLMs to established CDS tools represents a novel use of LLMs as a potential decision support tool in burn resuscitation.

**Funding for the study:**

N/A.

